# Development and validation of an explainable XGBoost model for early mortality prediction in pediatric sepsis

**DOI:** 10.3389/fped.2026.1933395

**Published:** 2026-08-31

**Authors:** Hong-mei Chen, Li-nong Wang, Yan-bing Fu, Jun Hua

**Affiliations:** Department of Emergency Medicine, Children's Hospital of Soochow University, Suzhou, China

**Keywords:** explainable artificial intelligence (SHAP), machine learning, mortality prediction, pediatric sepsis, PICU (pediatric intensive care unit), XGBoost

## Abstract

**Objective:**

To develop and internally validate an explainable machine learning model for early mortality prediction in pediatric sepsis using routinely available clinical variables, to support clinical risk stratification and decision-making.

**Methods:**

A total of 752 children with sepsis admitted to the Pediatric Intensive Care Unit (PICU) at the Children's Hospital of Soochow University between January 1, 2019, and June 30, 2023, were retrospectively enrolled. Twenty-six routinely available clinical variables obtained within 24 h after PICU admission were included. After data preprocessing, variables with excessive missing values, significant multicollinearity, or limited univariate predictive value were excluded. Clinically meaningful ratio-derived variables were constructed, and logarithmic transformation was applied to selected right-skewed continuous variables. Twelve supervised machine learning algorithms were developed and compared for mortality prediction. Model performance was evaluated using the area under the receiver operating characteristic curve (AUC), precision–recall (PR) curve, decision curve analysis (DCA), and other performance metrics. Model interpretability was assessed using SHapley Additive exPlanations (SHAP).

**Results:**

Among the 12 machine learning algorithms, extreme gradient boosting (XGBoost) achieved the best overall predictive performance. Following recursive feature elimination based on permutation importance, a simplified XGBoost model incorporating seven variables was established and interpreted using SHAP. In the internal validation cohort, the final model achieved an AUC of 0.803, with a sensitivity of 0.846, specificity of 0.696, accuracy of 0.722, and an F1 score of 0.512. The mean AUCs obtained from 5-fold and 10-fold cross-validation were 0.780 ± 0.044 and 0.771 ± 0.070, respectively, indicating good model stability. SHAP analysis identified pH, PaO₂, glucose, blood urea nitrogen (BUN), lactate dehydrogenase (LDH), international normalized ratio (INR), and the BUN-to-creatinine ratio (BUN/Cr) as the features that made the greatest contributions to the final model's mortality predictions.

**Conclusion:**

An explainable XGBoost-based prediction model was developed for early mortality prediction in pediatric sepsis using seven routinely available clinical variables collected within 24 h after PICU admission. The model demonstrated good discrimination, stability, and model-based interpretability, suggesting its potential as a practical tool for early risk stratification and individualized clinical management. Further multicenter external validation is warranted before routine clinical implementation.

## 1 Introduction

Sepsis and septic shock remain major challenges in pediatric emergency and critical care medicine because of their rapid progression and high mortality. Despite advances in intensive care, pediatric sepsis continues to impose a substantial clinical and healthcare burden worldwide. Relevant statistics show that the global mortality rate of pediatric sepsis ranges from 4% to 50%. The disease can change rapidly, and death often occurs within the first 2–3 days of treatment (1, 2). Early diagnosis and timely, appropriate resuscitation and treatment are essential for improving prognosis.

Several clinical scoring systems, including PRISM III, PELOD-2, pSOFA, and the recently proposed Phoenix Sepsis Score, are widely used to assess illness severity, organ dysfunction, or diagnose pediatric sepsis (3–6). However, these scoring systems were not specifically developed for individualized early mortality prediction. Moreover, some require numerous clinical variables, repeated assessments, or relatively complex calculations, which may reduce their practicality for rapid bedside risk stratification in critically ill children. Therefore, a simple and accurate prediction model based on routinely available clinical variables may provide complementary value for early mortality risk assessment.

Recent advances in machine learning have demonstrated considerable potential for improving clinical prediction by capturing complex nonlinear relationships among clinical variables. Several recent studies have developed machine-learning models based on XGBoost, LightGBM, CatBoost, random forest, and meta-ensemble learning for sepsis prediction and clinical decision-support applications, demonstrating improved predictive performance over conventional statistical approaches (7–9). More recently, explainable artificial intelligence (XAI) techniques, particularly SHapley Additive exPlanations (SHAP), have been increasingly incorporated into machine-learning models to improve transparency, facilitate clinical interpretation, and enhance clinician trust in artificial intelligence-assisted decision-making (10, 11). Although these studies were conducted in different clinical scenarios, they consistently demonstrate that improving model interpretability is becoming an important prerequisite for the clinical adoption of artificial intelligence (12–14).

Existing pediatric machine-learning models often rely on numerous predictors, lack explainability, or have not been translated into clinically deployable tools. Nevertheless, most existing machine-learning studies have focused on adult populations, early sepsis detection rather than mortality prediction, or models requiring large numbers of electronic health record variables, thereby limiting their applicability in pediatric critical care. Furthermore, few studies have simultaneously emphasized model simplicity, predictive performance, interpretability, and bedside implementation specifically for pediatric sepsis. Therefore, the present study aimed to develop and internally validate an explainable XGBoost-based model for early mortality prediction in children with sepsis using routinely available clinical variables collected within 24 h after PICU admission. Compared with existing pediatric sepsis prediction models and conventional clinical scoring systems, the proposed model was designed to balance predictive performance, interpretability, and clinical feasibility by using only seven routinely available clinical variables, maintaining robust discrimination after feature reduction, incorporating SHAP-based explanations, and providing a web-based application for bedside implementation and individualized risk assessment.

## 2 Materials and methods

### 2.1 General data

This retrospective study included 752 children with sepsis who were admitted to the Pediatric Intensive Care Unit (PICU) of the Children's Hospital of Soochow University between January 2019 and June 2023. Sepsis was diagnosed according to the Phoenix Sepsis Criteria (Phoenix Sepsis Score ≥2 in children with suspected infection) (3, 15). Children who were discharged within 2 h of admission because of incomplete clinical evaluation or who died within 2 h after admission were excluded. This study was approved by the hospital ethics committee (approval No. 2025CS037).

### 2.2 Data collection

Clinical data were extracted from the electronic medical record system. The primary outcome was in-hospital survival status (survival or death). Variables collected within the first 24 h after PICU admission included vital signs [pulse, respiratory rate, mean arterial pressure(map), and peripheral oxygen saturation (SpO₂)]; complete blood count parameters (white blood cell count [WBC], hemoglobin [Hb], platelet count [Plt], and neutrophil percentage [NeuPct]); inflammatory biomarkers [C-reactive protein [CRP], procalcitonin [PCT], and B-type natriuretic peptide [BNP]]; arterial blood gas and electrolyte parameters (pH, partial pressure of carbon dioxide [PCO₂], partial pressure of oxygen [PaO₂], actual bicarbonate [AB], base excess [BE], lactate [Lac], and sodium [Na]); biochemical indices (albumin [ALB], calcium [Ca], glucose [Glu], blood urea nitrogen [BUN], creatinine [Cr], and lactate dehydrogenase [LDH]); and coagulation indicators [international normalized ratio [INR] and activated partial thromboplastin time [APTT]]. For each variable, the most abnormal value recorded during the first 24 h after PICU admission was used for analysis.

### 2.3 Statistical methods

All statistical analyses and machine learning procedures were performed using Python 3.10.11 (https://www.python.org). The dataset was randomly divided into a training cohort (80%, *n* = 601) and an internal validation cohort (20%, *n* = 151) using stratified sampling to preserve the outcome distribution. The training cohort was used for data preprocessing, feature selection, hyperparameter optimization, and model development, whereas the internal validation cohort was reserved for independent performance evaluation.

#### 2.3.1 Data preprocessing

The proportion of missing values was first calculated for each variable and each patient. No patient was excluded because the proportion of missing variables exceeded 25%. At the variable level, BNP had a missing rate greater than 25% (466/752, 62.0%) and was therefore excluded. All remaining variables were retained for subsequent analyses.

Three clinically meaningful ratio-derived variables were subsequently constructed: the PaO₂/FiO₂ ratio (PaO₂_FiO₂_ratio), the blood urea nitrogen-to-creatinine ratio (BUN_Cr_ratio), and the neutrophil-to-lymphocyte percentage ratio (NeuPct_LymPct_ratio), representing oxygenation efficiency, renal function or circulatory perfusion status, and inflammatory-immune imbalance, respectively (16–18). Because several laboratory variables exhibited right-skewed distributions, logarithmic transformation [log(1 + x)] was applied to WBC, CRP, lactate, glucose, BUN, creatinine, and LDH. Both transformed and original variables were evaluated during feature screening, and the clinically more interpretable variable was retained if no obvious predictive improvement was observed after transformation.

Missing values were imputed using multiple imputation by chained equations (MICE) (19), implemented with the IterativeImputer algorithm and Bayesian Ridge regression. To avoid information leakage, the imputation model was fitted using the training set only and then applied to the internal validation set. Five imputed datasets were generated with a maximum of ten iterations, and the averaged values were used for subsequent analyses. After imputation, no missing values remained.

To minimize feature redundancy while preserving clinically informative predictors, feature selection was performed exclusively within the training set to avoid information leakage. A sequential strategy combining univariate ROC screening, multicollinearity assessment using VIF, and model-based recursive feature elimination (RFE) was adopted. This approach enabled the removal of weak predictors, reduction of redundant information, and identification of the smallest feature subset that maintained predictive performance while improving model interpretability.

First, preliminary feature screening was performed based on univariate predictive performance (20). Receiver operating characteristic (ROC) analysis was conducted for each candidate variable in the training set to identify variables with acceptable individual discriminatory ability. Both original and log(1 + x)-transformed variables were evaluated, and the clinically more interpretable original variable was retained when the transformed variable did not provide a meaningful improvement in predictive performance. After training-set-based univariate ROC screening, 18 candidate features were retained: MAP, SpO2, pH, PCO2, PaO2, AB, BE, Lac, Na, ALB, Ca, Glu, BUN, Cr, LDH, INR, APTT, and BUN_Cr_ratio. Removed variables included CRP, PCT, Hb, NeuPct, WBC, Resp, Plt, Pulse, PaO2_FiO2_ratio, NeuPct_LymPct_ratio, and transformed variables that did not yield clear predictive gains or had insufficient performance: log1p_Lac, log1p_LDH, log1p_BUN, log1p_Cr, log1p_Glu, log1p_CRP, and log1p_WBC. Details of the training-set-based univariate ROC screening and feature retention process are provided in Supplementary Table S1.

Second, variance inflation factor (VIF) analysis was performed in the training set to evaluate multicollinearity among candidate predictors and improve model stability. VIF analysis showed that BE exhibited substantial multicollinearity (VIF = 30.161) and was therefore excluded. All remaining variables had acceptable VIF values (<10) (21), as shown in Supplementary Table S2. Finally, 17 variables were retained for model development: MAP, SpO₂, pH, PCO₂, PaO₂, AB, Lac, Na, ALB, Ca, Glu, BUN, Cr, LDH, INR, APTT, and BUN_Cr_ratio.

Recursive feature elimination based on permutation importance was subsequently performed during model development to identify the smallest subset of predictors that preserved predictive performance while improving model simplicity. Overall, this sequential feature-selection strategy combined statistical screening, multicollinearity assessment, and model-based optimization to enhance model interpretability, reduce overfitting, and improve clinical applicability.

#### 2.3.2 Model development and feature selection

Twelve supervised machine learning algorithms were developed for mortality prediction, including Random Forest (RF), Adaptive Boosting (AdaBoost), Extreme Gradient Boosting (XGBoost) (22), Gradient Boosting Machine (GBM), Artificial Neural Network (ANN), Decision Tree (DT), Extra Trees (EXT), Logistic Regression (GLM), K-Nearest Neighbors (KNN), Naïve Bayes (NB), Support Vector Machine (SVM), and Light Gradient Boosting Machine (LightGBM). For each algorithm, 5-fold stratified cross-validation combined with grid search was performed in the training set for hyperparameter tuning, with ROC-AUC as the optimization metric.

First,full models incorporating all 17 candidate variables were developed and evaluated in the internal validation cohort using ROC-AUC, sensitivity, specificity, precision, accuracy, and F1 score. Models were then preliminarily screened according to internal validation AUC: The four best-performing algorithms (XGBoost, NB, ANN, and GLM) were subsequently selected for recursive feature elimination (23). In each iteration, permutation importance was calculated to quantify each variable's contribution to model performance (24), The least important variable was removed, and the model was retrained until only three variables remained. Considering both predictive performance and clinical applicability, the optimal model was selected from models containing five to eight variables. The final XGBoost model incorporated seven predictors: pH, PaO₂, Glu, BUN, LDH, INR, and BUN_Cr_ratio.

To provide a more robust estimate of model performance beyond a single train–test split, model robustness was further assessed using five-fold and ten-fold stratified cross-validation together with repeated stratified random split sensitivity analysis. Nested cross-validation was not adopted because the primary objective of this study was model development and internal validation using an independent hold-out validation cohort. To further evaluate robustness beyond a single train-test partition, repeated stratified random split sensitivity analysis was additionally performed. The full dataset was randomly divided 100 times into training and internal validation sets using an 80:20 stratified sampling strategy while preserving the outcome distribution. Feature selection was not repeated during these analyses. Instead, the final seven predictors and the optimized XGBoost hyperparameters identified in the primary analysis were fixed, and only model fitting and validation were repeated. For each repetition, the XGBoost model was trained using the training subset and evaluated in the corresponding validation subset. The mean, standard deviation, and 2.5th–97.5th percentile intervals of ROC-AUC, PR-AUC, Brier score, sensitivity, specificity, accuracy, and F1 score were calculated across all repetitions. The complete results of the repeated stratified random split sensitivity analysis are provided in Supplementary Table S3.

Because mortality accounted for only 16.9% of the study cohort, model performance was evaluated using precision–recall AUC (PR-AUC), sensitivity, specificity, F1 score, and decision curve analysis (DCA), in addition to ROC-AUC. To evaluate the potential impact of class imbalance, additional sensitivity analyses were performed using balanced sample weighting and the XGBoost scale_pos_weight parameter. Synthetic Minority Over-sampling Technique (SMOTE) was not adopted in the primary analysis because synthetic oversampling in a relatively modest-sized clinical dataset may generate artificial patient profiles, distort the underlying data distribution, and increase the risk of model overfitting. The results of the class-imbalance sensitivity analyses are provided in Supplementary Table S4.

To contextualize model performance, the final XGBoost model was further compared with established pediatric clinical severity scores, including PRISM III and PELOD-2. ROC-AUC and PR-AUC were calculated for each scoring system. For scores in which lower values indicated higher mortality risk, score orientation was reversed before calculating discrimination metrics. The complete comparison with available clinical severity scores is provided in Supplementary Table S5.

Receiver operating characteristic (ROC) curves, precision-recall (PR) curves, and decision curve analysis (DCA) (25) were performed to comprehensively evaluate discrimination, precision, and potential clinical utility. Calibration was assessed in the internal validation cohort using calibration curves and the Brier score to evaluate agreement between predicted and observed mortality probabilities. As shown in Supplementary Figure S1 and Supplementary Table S6, the calibration curve and grouped calibration data demonstrated reasonable agreement between the predicted probabilities and observed mortality rates. The optimal probability threshold was determined using the Youden index because it provides an objective balance between sensitivity and specificity during model development. We acknowledge that the optimal threshold may vary according to clinical priorities. Therefore, threshold sensitivity analyses were additionally performed by evaluating multiple probability cutoffs and reporting the corresponding sensitivity and specificity to facilitate application in different clinical scenarios. The complete threshold sensitivity analysis is provided in Supplementary Table S7, and the sensitivity–specificity trends are shown in Supplementary Figure S2.

For reproducibility, all stochastic procedures were controlled using a fixed random seed (20251112), including data partitioning, model training, cross-validation, and bootstrap resampling. The optimal hyperparameters of all 12 candidate models, including the final XGBoost model, are provided in Supplementary Supplementary Table S8. The Python version, package dependencies, and random seed are provided in Supplementary Supplementary Table S9. Bootstrap resampling with 1,000 iterations was performed to estimate 95% confidence intervals for discrimination, calibration, and classification performance metrics in the internal validation cohort. The bootstrap confidence intervals are provided in Supplementary Table S10.

#### 2.3.3 Model interpretation

To improve model transparency and facilitate clinical interpretation, SHapley Additive exPlanations (SHAP) were used to explain the final XGBoost model (10, 11).

Global interpretability was assessed using SHAP summary plots and bar plots based on the mean absolute SHAP value of each feature. Beeswarm plots illustrated the distribution of SHAP values across all patients, where each point represented an individual patient. Positive SHAP values indicated that a feature moved the model output toward the mortality class, whereas negative SHAP values moved the model output toward the survival class. These values represent model-based predictive contributions and should not be interpreted as causal or protective effects. SHAP dependence plots were further generated to explore the relationships between continuous predictor values and their corresponding SHAP values.

For individual-level interpretation, representative survival and death cases from the validation cohort were visualized using SHAP waterfall plots to illustrate how each predictor contributed to the final prediction.

Therefore, SHAP-based feature importance should not be interpreted as evidence that a predictor independently causes or prevents mortality.

#### 2.3.4 Model deployment and clinical application interface

To facilitate clinical application, a web-based pediatric sepsis mortality prediction tool was developed using the Streamlit framework.

The application automatically loads the trained XGBoost model and corresponding feature set. Users enter seven routinely available clinical variables (pH, PaO₂, Glu, BUN, Cr, LDH, and INR), after which the BUN/Cr ratio is calculated automatically. The system then estimates the individual probability of in-hospital mortality and classifies patients into high-risk or low-risk groups according to the optimal probability threshold determined in the internal validation cohort.

## 3 Results

### 3.1 Patient characteristics

A total of 752 children with sepsis were included in this study for model development and internal validation, comprising 625 survivors (83.1%) and 127 non-survivors (16.9%). The dataset was randomly divided into a training cohort (80%, *n* = 601) and an internal validation cohort (20%, *n* = 151) using stratified sampling to preserve the outcome distribution. The training cohort included 500 survivors and 101 non-survivors, whereas the internal validation cohort included 125 survivors and 26 non-survivors. The mortality rates were 16.8% in the training cohort and 17.2% in the internal validation cohort, indicating a comparable distribution of outcomes between the two cohorts. Baseline demographic and clinical characteristics of the survival and non-survival groups are summarized in Table 1.

**Table 1:** Baseline demographic and clinical characteristics of the survival and non-survival groups.

variable	survival	death	*p*_value
INR, dimensionless	1.230 [1.100, 1.440]	1.630 [1.275, 2.270]	<0.001
AB, mmol/L	20.300 [16.800, 23.000]	15.300 [10.850, 20.750]	<0.001
BE, mmol/L	−3.300 [−6.800, −0.300]	−8.300 [−14.050, −1.600]	<0.001
Lac, mmol/L	2.400 [1.600, 4.300]	4.500 [2.150, 10.800]	<0.001
log1p_Lac, dimensionless	1.224 [0.956, 1.668]	1.705 [1.147, 2.468]	<0.001
LDH, U/L	419.300 [301.900, 673.500]	745.800 [392.600, 1403.350]	<0.001
log1p_LDH, dimensionless	6.041 [5.713, 6.514]	6.616 [5.975, 7.247]	<0.001
pH, dimensionless	7.430 [7.360, 7.500]	7.330 [7.195, 7.470]	<0.001
log1p_Cr, dimensionless	3.520 [3.186, 3.865]	3.807 [3.487, 4.396]	<0.001
Cr, μmol/L	32.800 [23.200, 46.700]	44.000 [31.700, 80.100]	<0.001
BUN, mmol/L	4.430 [3.230, 5.920]	5.710 [4.360, 8.530]	<0.001
log1p_BUN, dimensionless	1.692 [1.442, 1.934]	1.904 [1.679, 2.254]	<0.001
APTT, s	34.300 [28.500, 41.900]	42.500 [31.700, 65.400]	<0.001
PaO2, mmHg	131.000 [89.600, 150.000]	150.000 [126.500, 161.500]	<0.001
SpO2, %	98.000 [96.000, 99.000]	96.000 [90.000, 98.000]	<0.001
ALB, g/L	39.600 [35.500, 43.200]	36.500 [32.050, 40.950]	<0.001
Ca, mmol/L	1.060 [0.990, 1.130]	1.010 [0.935, 1.080]	<0.001
Glu, mmol/L	6.100 [5.000, 7.700]	7.300 [5.200, 12.850]	<0.001
log1p_Glu, dimensionless	1.960 [1.792, 2.163]	2.116 [1.825, 2.628]	<0.001
MAP, mmHg	78.000 [68.000, 89.000]	74.000 [58.500, 86.500]	0.00567
BUN_Cr_ratio, dimensionless	0.136 [0.097, 0.177]	0.120 [0.092, 0.155]	0.0168
Na, mmol/L	136.000 [133.000, 139.000]	138.000 [133.000, 141.000]	0.0201
PCO2, mmHg	29.600 [23.900, 36.100]	26.600 [22.750, 34.700]	0.0249
log1p_CRP, dimensionless	2.338 [0.990, 4.024]	1.975 [0.878, 3.819]	0.224
CRP, mg/L	9.360 [1.690, 54.950]	6.210 [1.405, 44.570]	0.224
Hb, g/L	101.000 [83.000, 118.000]	103.000 [81.500, 125.000]	0.282
NeuPct_LymPct_ratio, dimensionless	2.282 [0.550, 5.964]	1.559 [0.520, 5.297]	0.425
PCT, ng/mL	0.580 [0.160, 8.626]	0.660 [0.115, 20.012]	0.454
Pulse, bpm	138.000 [117.000, 164.000]	133.000 [111.000, 170.000]	0.637
PaO2_FiO2_ratio, dimensionless	424.000 [282.381, 610.345]	405.000 [255.960, 639.429]	0.678
Plt, × 10^9^/L	156.000 [40.000, 291.000]	153.000 [60.000, 274.500]	0.729
Resp, bpm	30.000 [23.000, 40.000]	30.000 [23.000, 40.000]	0.824
NeuPct, %	61.700 [28.600, 80.400]	55.700 [32.600, 78.950]	0.869
WBC, × 10^9^/L	9.830 [5.130, 17.150]	10.210 [4.650, 17.535]	0.916
log1p_WBC, dimensionless	2.382 [1.813, 2.899]	2.417 [1.732, 2.920]	0.916

### 3.2 Model development and comparison

**Figure 1:**
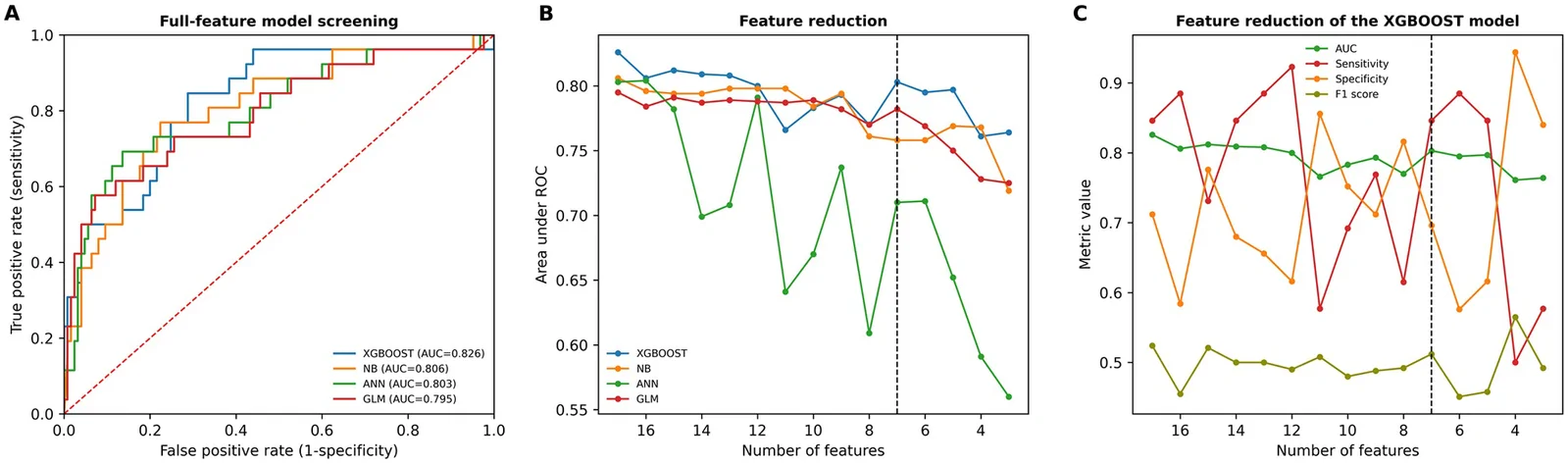
Performance comparison of machine learning models for predicting mortality in children with sepsis. **(A)** Receiver operating characteristic (ROC) curves of the four best-performing machine learning models. **(B)** Changes in the area under the ROC curve (AUC) of the selected models with decreasing numbers of features during recursive feature elimination. **(C)** Predictive performance of the XGBoost model across different feature subsets. NB, naive Bayes; GLM, generalized linear model; XGBoost, extreme gradient boosting; ANN, artificial neural network; ROC, receiver operating characteristic; AUC, area under the receiver operating characteristic curve.

Using the training cohort, 12 supervised machine learning algorithms were developed to predict in-hospital mortality in children with sepsis. Model performance was subsequently evaluated in the independent internal validation cohort. Among all models, the extreme gradient boosting (XGBoost) model achieved the highest discriminative performance, with an AUC of 0.826, followed by the naive Bayes (NB) model (AUC = 0.806), artificial neural network (ANN) model (AUC = 0.803), and generalized linear model (GLM) (AUC = 0.795). Receiver operating characteristic (ROC) curves for these four best-performing models are presented in Figure 1A.

Recursive feature elimination was subsequently performed for the four selected models. Across different feature subsets, the XGBoost model consistently demonstrated superior or near-superior predictive performance. The optimal balance between model performance and clinical simplicity was achieved with seven features, yielding an AUC of 0.803 in the internal validation cohort (Figures 1B,C). Accordingly, XGBoost was selected as the final prediction model. The final model incorporated seven routinely available clinical variables: pH, PaO₂, glucose (Glu), blood urea nitrogen (BUN), lactate dehydrogenase (LDH), international normalized ratio (INR), and the blood urea nitrogen-to-creatinine ratio (BUN/Cr).

### 3.3 Final model

The final prediction model was determined through the recursive feature elimination process of the XGBoost algorithm. The initial full-feature model incorporated 17 candidate variables and achieved an AUC of 0.826 in the internal validation cohort. As variables were sequentially removed, the model maintained relatively stable discriminative performance.

**Figure 2:**
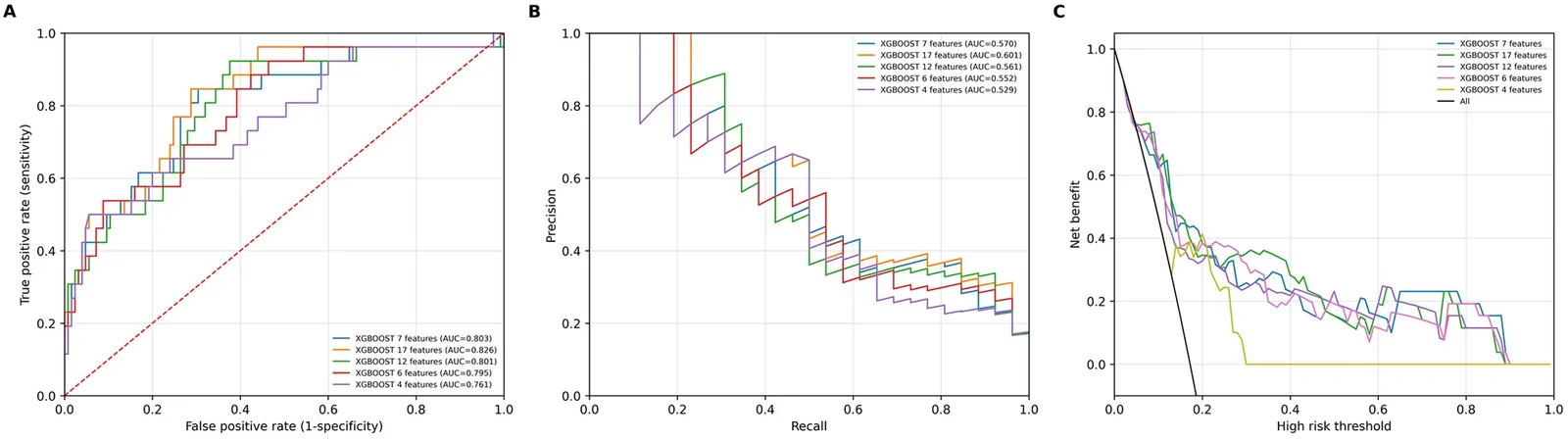
Comparison of the predictive performance of XGBoost models with different numbers of features. **(A)** Receiver operating characteristic (ROC) curves. **(B)** Precision–recall (PR) curves. **(C)** Decision curve analysis (DCA) curves.

Compared with the full-feature model, the seven-feature XGBoost model achieved an AUC of 0.803, with no statistically significant difference according to the DeLong test (*P* = 0.228), indicating that a substantial reduction in the number of variables was achieved without compromising predictive performance (40). However, when the feature set was further reduced to four variables, the AUC decreased to 0.761, which was significantly lower than that of the full-feature model (*P* = 0.041), suggesting that excessive feature reduction impaired model discrimination. Comparisons of the ROC, precision–recall (PR), and decision curve analysis (DCA) curves for models with different numbers of features are presented in Figure 2. The seven-feature model achieved an optimal balance between predictive performance and model parsimony while maintaining good clinical applicability. Its area under the PR curve was 0.570, slightly higher than that of the 12-feature model (0.561) and comparable to that of the full-feature model, indicating favorable positive predictive performance and potential clinical utility for mortality prediction.

The final model incorporated seven routinely available clinical variables: pH, PaO₂, glucose (Glu), blood urea nitrogen (BUN), lactate dehydrogenase (LDH), international normalized ratio (INR), and the blood urea nitrogen-to-creatinine ratio (BUN/Cr). The final XGBoost model was trained using the optimized hyperparameters identified through grid search: n_estimators = 50, max_depth = 2, learning_rate = 0.1, subsample = 0.7, colsample_bytree = 1.0, min_child_weight = 5, and reg_lambda = 5. The complete hyperparameter configurations of all evaluated models are provided in Supplementary Table S8.

In the internal validation cohort, the final XGBoost model achieved an AUC of 0.803(95% CI, 0.683–0.893) for predicting in-hospital mortality, with a sensitivity of 0.846(95% CI, 0.692–0.964), specificity of 0.696(95% CI, 0.619–0.771), accuracy of 0.722(95% CI, 0.649–0.788), F1 score of 0.512(95% CI, 0.369–0.633), and an optimal probability cutoff of 0.121. Threshold sensitivity analysis showed that sensitivity decreased and specificity increased as the prediction probability threshold increased. At thresholds of 0.05, 0.20, and 0.50, the sensitivities were 0.962, 0.577, and 0.346, respectively, whereas the corresponding specificities were 0.136, 0.840, and 0.960. The complete threshold sensitivity analysis is presented in Supplementary Table S7, and the sensitivity-specificity trends are shown in Supplementary Figure S2. These findings suggest that the probability threshold can be adjusted according to different clinical priorities. Calibration analysis demonstrated good agreement between predicted and observed mortality probabilities, with a Brier score of 0.107, The corresponding calibration curve is shown in Supplementary Figure S1. In class-imbalance sensitivity analyses, the original final XGBoost model achieved an AUC of 0.803, PR-AUC of 0.570, Brier score of 0.107, sensitivity of 0.846, specificity of 0.696, and F1 score of 0.512. The balanced sample-weight model achieved a similar AUC of 0.802 and higher sensitivity of 0.885, but lower specificity of 0.664, lower PR-AUC of 0.554, and worse Brier score of 0.157. The model using scale_pos_weight also showed a similar AUC of 0.802 but lower PR-AUC of 0.542 and worse Brier score of 0.160. Overall, imbalance-handling strategies did not improve overall predictive performance or calibration and therefore were not incorporated into the final model. The complete results are shown in Supplementary Table S4.

**Figure 3:**
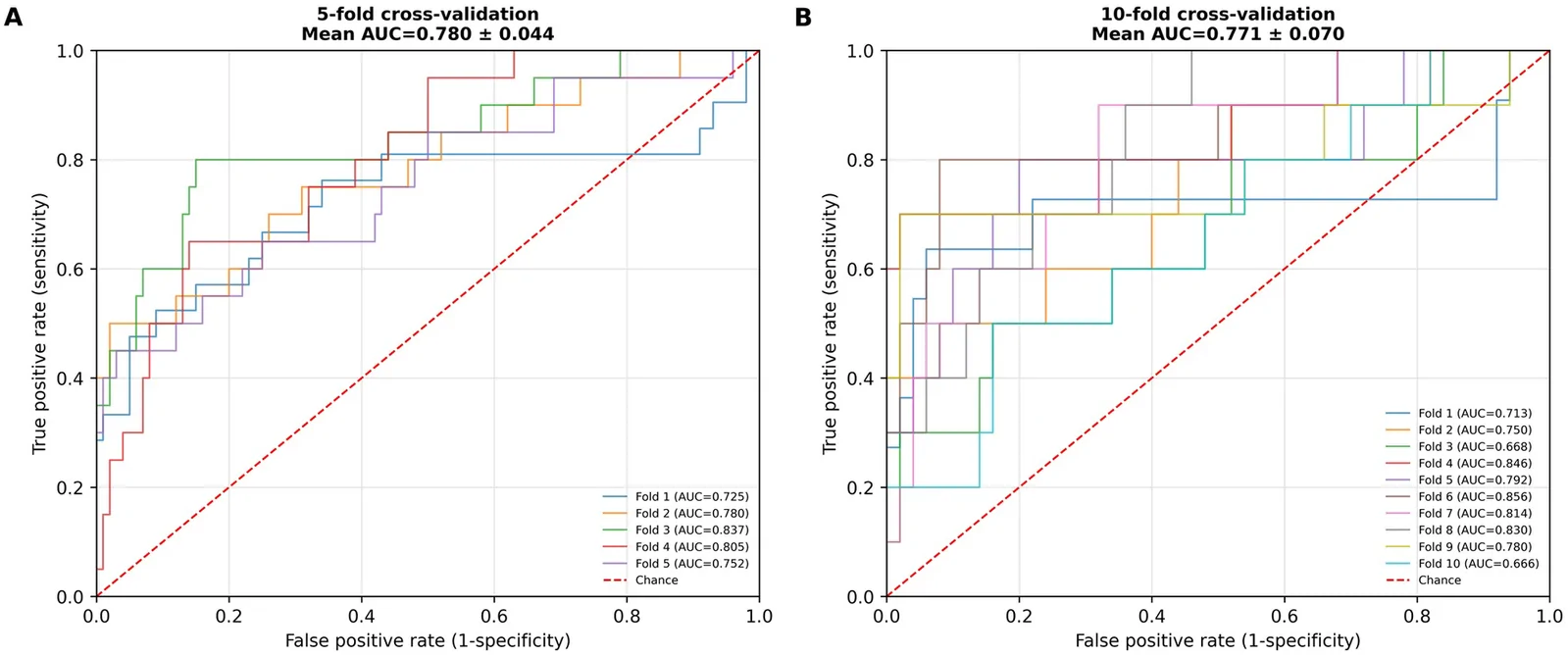
Internal validation of the final XGBoost model using stratified cross-validation. **(A)** Receiver operating characteristic (ROC) curves from five-fold stratified cross-validation. **(B)** Receiver operating characteristic (ROC) curves from ten-fold stratified cross-validation.

To further assess model robustness, five-fold and ten-fold stratified cross-validation were performed in the training cohort, yielding mean AUCs of 0.780 ± 0.044 and 0.771 ± 0.070, respectively. The corresponding ROC curves are shown in Figure 3.

To further evaluate the stability of model performance across different data partitions, a repeated 80:20 stratified random split sensitivity analysis was performed. Across 100 repetitions, the final seven-feature XGBoost model showed stable performance, with a mean AUC of 0.780 ± 0.053 and a 2.5th to 97.5th percentile range of 0.668–0.875. The mean PR-AUC was 0.584 ± 0.079, and the mean Brier score was 0.106 ± 0.011. The mean sensitivity and specificity were 0.672 ± 0.128 and 0.823 ± 0.106, respectively. These findings indicate that the predictive performance of the final model was robust across different random data partitions and was not dependent on a single favorable train-test split. The complete results of the repeated stratified random split sensitivity analysis are provided in Supplementary Table S3.

**Figure 4:**
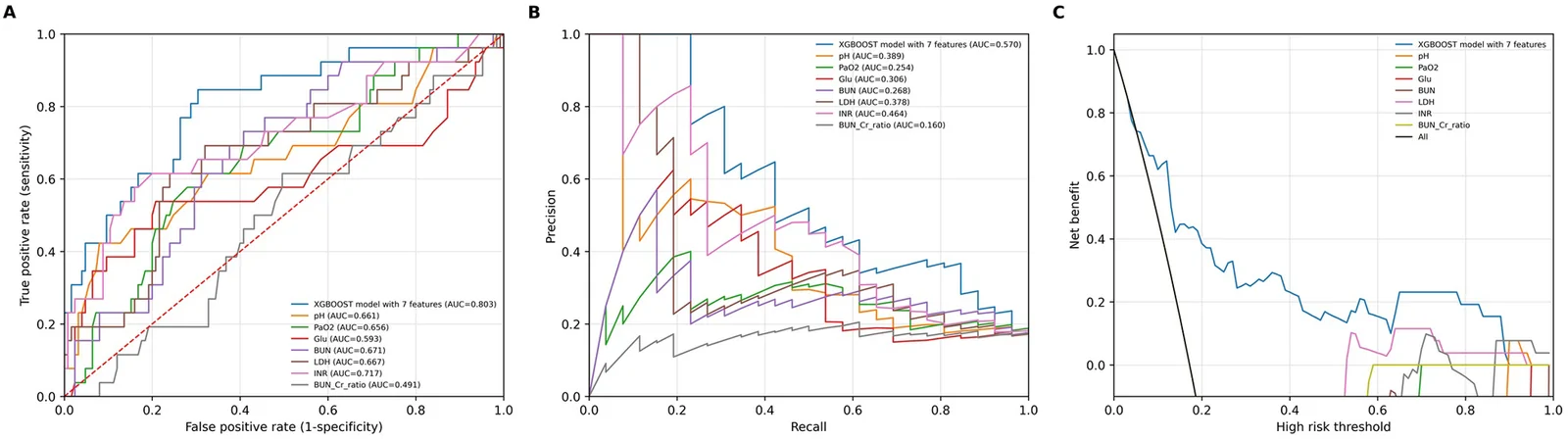
Comparison of the predictive performance of the final XGBoost model with the individual predictors included in the model. **(A)** Receiver operating characteristic (ROC) curves. **(B)** Precision–recall (PR) curves. **(C)** Decision curve analysis (DCA) curves.

The predictive performance of the final seven-feature XGBoost model was further compared with that of each individual predictor included in the model, namely pH, PaO₂, glucose (Glu), blood urea nitrogen (BUN), lactate dehydrogenase (LDH), international normalized ratio (INR), and the BUN-to-creatinine ratio (BUN/Cr). As shown in Figure 4, the final model achieved an AUC of 0.803 in the internal validation cohort, outperforming all individual predictors. Among the single variables, INR showed the highest discriminative ability (AUC = 0.717), followed by BUN (0.671), LDH (0.667), pH (0.661), PaO₂ (0.656), Glu (0.593), and BUN/Cr (0.491).

DeLong tests demonstrated that the final model achieved significantly higher AUCs than the single-variable models based on pH (AUC = 0.661, *P* = 0.0227), PaO₂ (AUC = 0.656, *P* = 0.0349), Glu (AUC = 0.593, *P* = 0.00186), BUN (AUC = 0.671, *P* = 0.0239), LDH (AUC = 0.667, *P* = 0.0307), BUN/Cr (AUC = 0.491, *P* < 0.001) and INR(AUC = 0.717, *P* = 0.048).

The final XGBoost model was further compared with available clinical severity scores. In the internal validation cohort, the final model achieved an AUC of 0.803 and PR-AUC of 0.570. The AUCs of PRISM III, and PELOD-2 were 0.828, and 0.827, respectively. PRISM III and PELOD-2 achieved slightly higher discrimination than the proposed model. However, the differences were modest. However, both scoring systems require substantially more clinical variables and were not specifically designed as simplified individualized mortality prediction tools. In contrast, the proposed model achieved competitive discrimination using only seven routinely available variables while additionally providing individualized SHAP-based explanations and web-based implementation. The complete comparison is shown in Supplementary Table S5.

Precision–recall analysis further demonstrated the superiority of the multivariable model. The final XGBoost model achieved a PR-AUC of 0.570, exceeding that of all individual predictors, indicating improved identification of patients at high risk of mortality. Decision curve analysis likewise showed that the final model provided greater net clinical benefit than any single predictor across a broad range of clinically relevant threshold probabilities, supporting its potential utility for early mortality risk stratification in pediatric sepsis.

The final XGBoost model was trained using the selected hyperparameters from grid search. The key hyperparameters were n_estimators = 50, max_depth = 2, learning_rate = 0.1, subsample = 0.7, colsample_bytree = 1.0, min_child_weight = 5, and reg_lambda = 5.

### 3.4 Model interpretation

**Figure 5:**
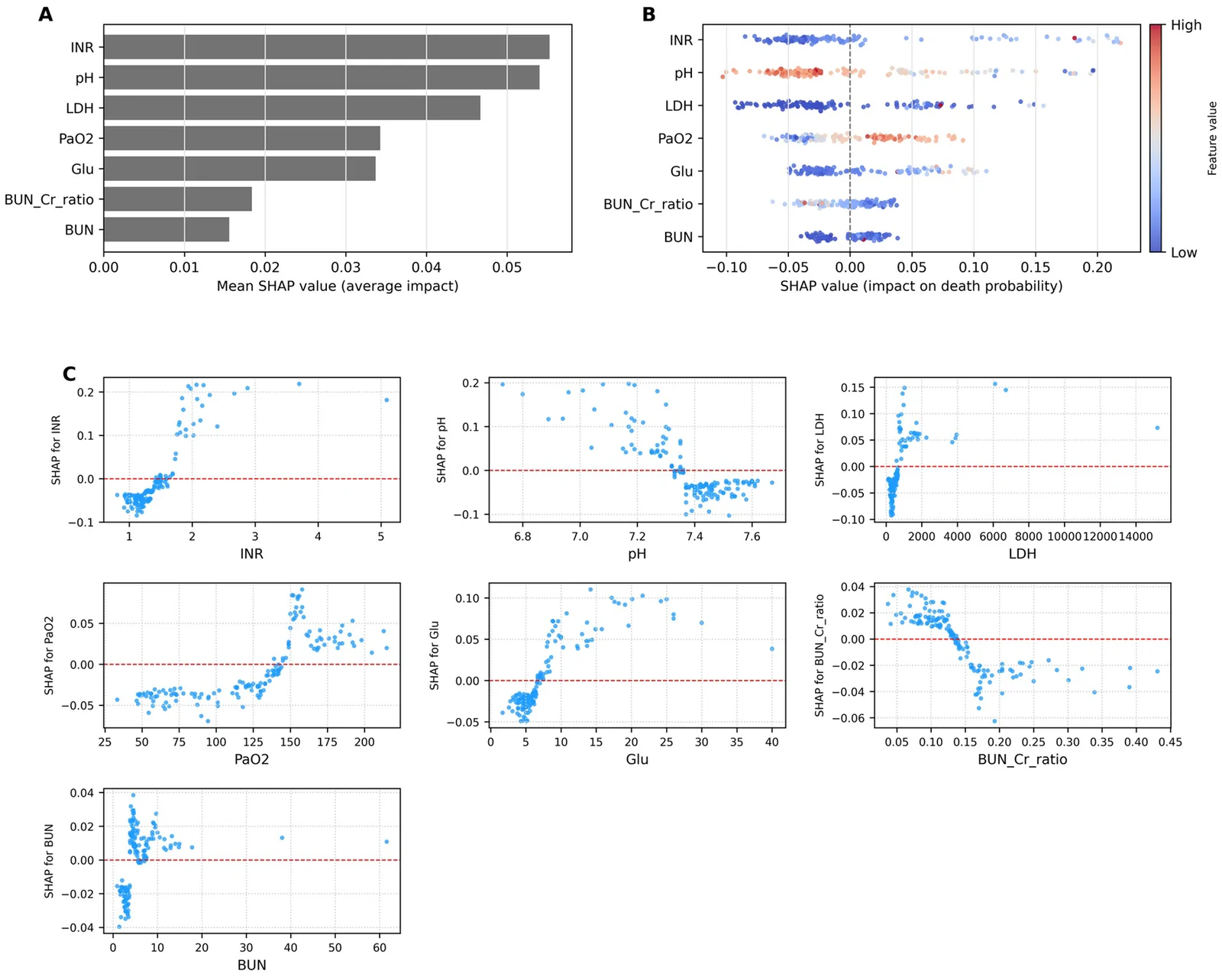
Global interpretation based on the SHAP method. **(A)** Mean absolute SHAP value bar plot showing the overall importance of each feature. **(B)** SHAP beeswarm plot illustrating the distribution of SHAP values for individual patients. Each point represents one patient, with color indicating the feature value (blue, low; red, high). **(C)** SHAP dependence plots showing the relationships between feature values and their contributions to the predicted mortality risk.

Because clinicians may find prediction models that cannot be directly interpreted difficult to accept, this study used SHAP to interpret the output of the final XGBoost model. SHAP provides both global feature-level interpretation and local individual-level interpretation by calculating the marginal contribution of each variable to model predictions. Global interpretation describes the overall prediction behavior of the model. As shown in the SHAP summary plots (Figures 5A,B), this study used mean absolute SHAP values to assess the overall contribution of each variable to model output and displayed them in descending order of importance.

The SHAP beeswarm plot further shows the relationship between each variable's value level and the direction of mortality risk prediction. Each point represents one child, the *x*-axis is the SHAP value, and the color indicates the actual value of the variable. A SHAP value greater than 0 indicates that the variable contributes to shifting the model output toward the mortality class, whereas a SHAP value less than 0 indicates that shifts the model output toward the survival class. The SHAP dependence plot (Figure 5C) was used to further observe the relationship between the actual value of a single variable and its SHAP value, helping to characterize how the model output varies across different observed levels of each variable.

**Figure 6:**
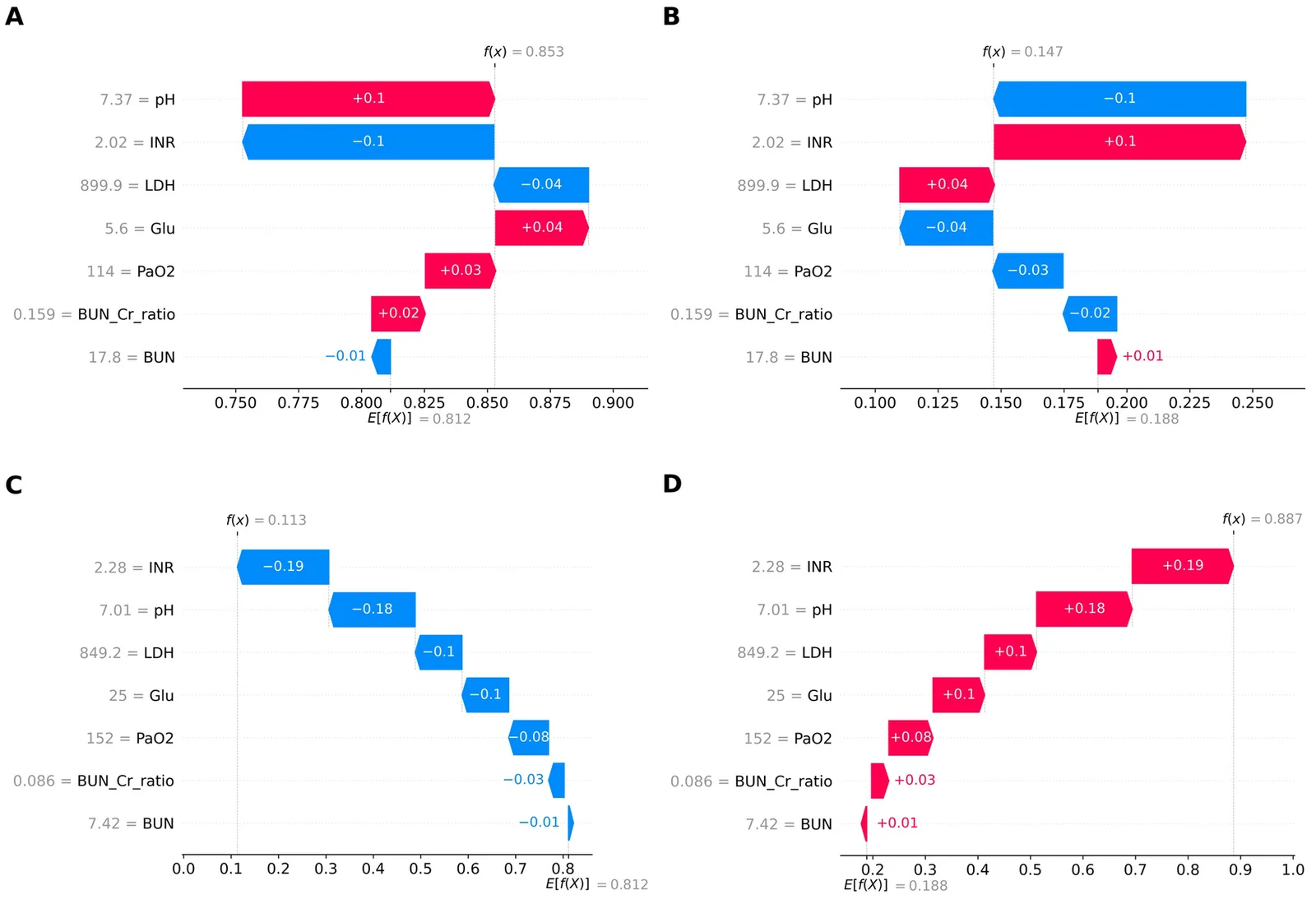
Local interpretation of the final XGBoost model using SHAP waterfall plots. **(A,B)** Representative survivor from the internal validation cohort with a predicted mortality probability of 14.7% (survival probability, 85.3%). **(C,D)** Representative non-survivor with a predicted mortality probability of 88.7% (survival probability, 11.3%). Red bars indicate features that increase the probability of the displayed outcome, whereas blue bars indicate features that decrease it.

This study used SHAP waterfall plots for local individual-level interpretation, showing how individual variables jointly increased or decreased the model-predicted probability of mortality for a specific child. This interpretation helps clinicians understand the model-based rationale for individual predictions, thereby improving transparency and acceptability in clinical application. As shown in Figures 6A,B, present the local interpretation results for a child in the internal validation set who did not actually die. The model predicted a mortality probability of 14.7% and a survival probability of 85.3%, indicating that the model judged this child as low risk. Figures 6C,D show a child in the internal validation set who actually died; the model predicted a mortality probability of 88.7% and a survival probability of 11.3%.

### 3.5 Clinical application

**Figure 7:**
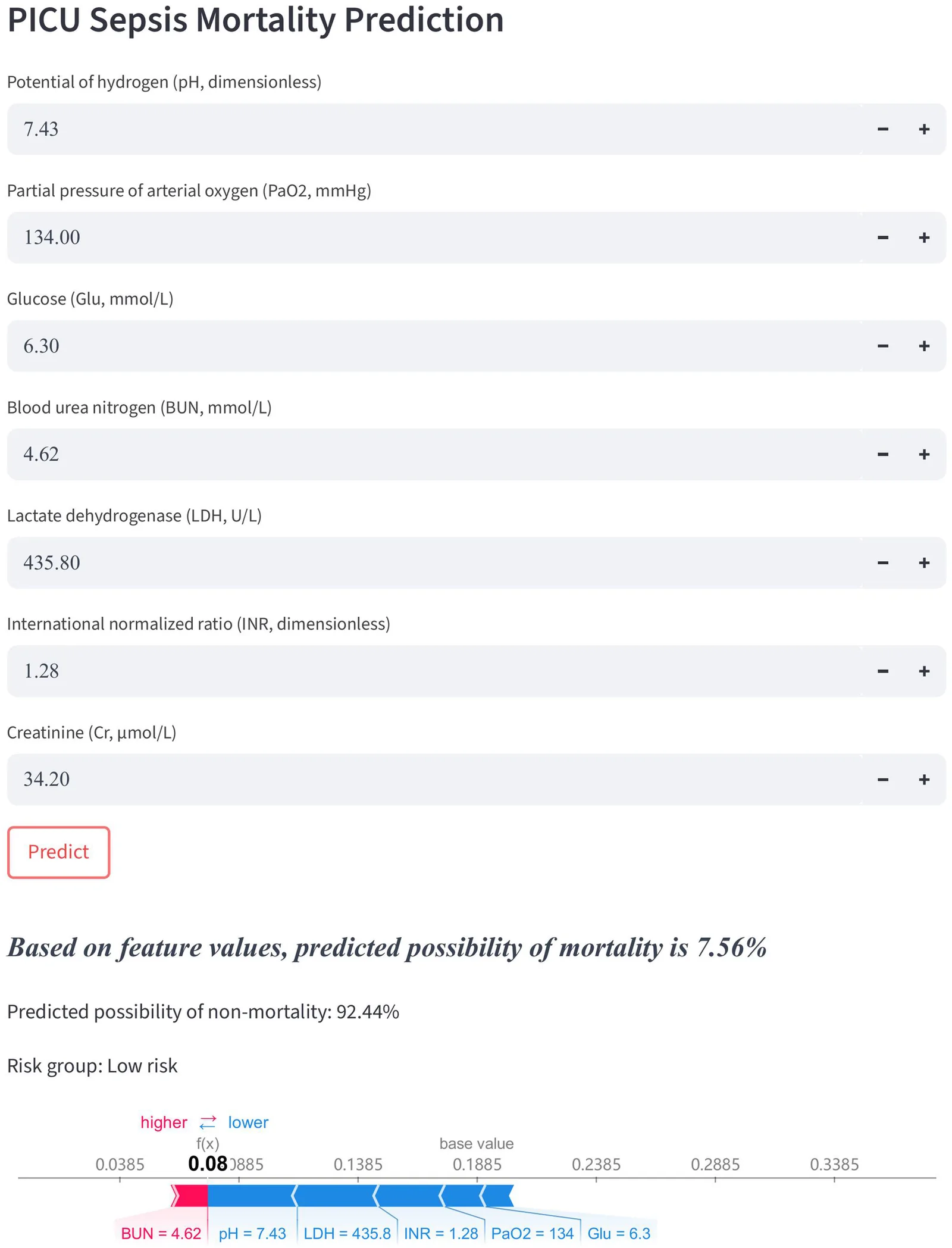
Web-based clinical application of the final XGBoost model. The application allows clinicians to enter routinely available clinical variables and automatically calculates the predicted mortality probability, survival probability, and risk category. SHAP-based local explanations are simultaneously displayed to illustrate the contribution of each variable to the individual prediction.

To facilitate clinical implementation, the final XGBoost model was deployed as a web-based application using the Streamlit framework (Figure 7). The application requires users to enter seven routinely available clinical variables, including pH, PaO₂, glucose (Glu), blood urea nitrogen (BUN), creatinine (Cr), lactate dehydrogenase (LDH), and international normalized ratio (INR). The BUN-to-creatinine ratio (BUN/Cr) is calculated automatically and incorporated into the prediction model.

After data entry, the application instantly generates the predicted probabilities of mortality and survival and classifies patients as high- or low-risk according to the optimal cutoff identified during internal validation. In addition, the application provides SHAP-based local explanations, visually illustrating the contribution of each variable to the predicted mortality risk for an individual patient. This interactive interface provides model-based explanations and may support individualized clinical risk assessment. It should be used as an adjunct to, rather than a replacement for, clinical judgment.

## 4 Discussion

As a common critical illness in children, pediatric sepsis has high incidence, high disability rates, and high mortality, and early identification and intervention are important for outcomes (26). Recently, international consensus updated the criteria for pediatric sepsis and septic shock by adopting the Phoenix Sepsis Score, aiming to identify life-threatening organ dysfunction caused by infection in children. However, in PICU clinical practice, children's conditions can change rapidly, and reliance on a single indicator or traditional scoring system alone may make it difficult to identify mortality risk in a timely and accurate manner. Therefore, constructing a risk prediction model based on routine clinical indicators with good predictive performance and interpretability has important clinical value.

The novelty of this study lies in the development of a simplified, explainable, and clinically deployable XGBoost model for early mortality prediction in pediatric sepsis. Unlike established clinical scoring systems that primarily quantify general illness severity or organ dysfunction, the proposed model was optimized for mortality risk prediction and incorporated nonlinear relationships among routine clinical variables. Furthermore, SHAP-based global and local explanations provided insight into how the model generated predictions at both population and individual levels, while the web application enabled rapid individualized risk estimation. The final model also showed superior predictive performance compared with individual predictors and demonstrated competitive performance relative to available clinical severity scores, supporting its potential value as a complementary decision-support tool rather than a replacement for established clinical assessment.

The present study is consistent with recent research emphasizing the value of explainable AI in clinical decision support. Compared with interpretable deep-learning models developed for image-based diagnosis and optimized ensemble methods used in other medical diagnostic tasks, our model was designed for a clinically specific tabular-data prediction problem: early mortality risk stratification in pediatric sepsis. By incorporating only seven routinely available variables and providing SHAP-based global and local explanations, the final model attempted to balance predictive performance, interpretability, and clinical practicality.

Based on clinical and laboratory indicators that are relatively easy to obtain within 24 h after PICU admission, this study used multiple machine learning algorithms to establish pediatric sepsis mortality risk prediction models and determined the final model through feature reduction, cross-validation, and SHAP interpretability analysis.

This study ultimately established a seven-feature XGBoost model including pH, PaO2, Glu, BUN, LDH, INR, and BUN_Cr_ratio. The model had an AUC of 0.803 in the internal validation set, indicating good discrimination for mortality in children with sepsis. Compared with single feature variables, the final model showed better overall predictive performance, suggesting that multidimensional integration of acid-base status, oxygenation status, metabolic status, renal function, tissue injury, and coagulation function helps more comprehensively reflect disease severity. Further 5-fold and 10-fold cross-validation showed mean AUCs of 0.780 ± 0.044 and 0.771 ± 0.070, respectively, indicating a certain degree of model stability.

Among the variables included in the final model, pH is an important indicator of systemic acid–base homeostasis and may reflect the severity of critical illness. In children with sepsis, acid–base disturbances commonly occur as a consequence of tissue hypoperfusion, lactate accumulation, and progressive organ dysfunction. As an integrated marker of metabolic and respiratory acid–base abnormalities, pH reflects the overall disturbance of the internal physiological environment. Previous studies have reported that lower pH levels are associated with increased in-hospital mortality in pediatric patients with sepsis, supporting its potential value for prognostic assessment (27).

PaO₂ reflects arterial oxygenation status and is widely used to evaluate respiratory function and oxygen delivery. In pediatric sepsis, impaired oxygenation may result from pulmonary infection, acute respiratory distress syndrome (ARDS), microcirculatory dysfunction, and impaired tissue oxygen utilization (16). Previous studies have demonstrated that oxygenation impairment, particularly reduced PaO₂/FiO₂ ratios, is associated with organ dysfunction and mortality in critically ill patients (4). Although PaO₂ alone represents only one aspect of oxygenation status, its inclusion as an input variable in our machine learning model is clinically plausible because it reflects underlying respiratory dysfunction and systemic severity.

Glucose (Glu) represents the metabolic stress response during critical illness. In sepsis, inflammatory activation, catecholamine surge, and insulin resistance contribute to abnormal glucose metabolism (28). Stress-induced hyperglycemia and glycemic variability have been associated with adverse outcomes in critically ill patients and may reflect the severity of systemic metabolic disturbances (29). The identification of Glu as an important feature in our model highlights the potential contribution of metabolic stress responses to mortality prediction in pediatric sepsis.

Blood urea nitrogen (BUN) and the BUN-to-creatinine ratio (BUN/Cr ratio) provide information regarding renal function, circulatory perfusion, and metabolic status. Sepsis-associated acute kidney injury (AKI) is a major determinant of prognosis, involving mechanisms such as impaired renal perfusion, inflammatory injury, and microcirculatory dysfunction (30, 31). Elevated BUN levels have been associated with increased severity of neonatal sepsis and independently predicted mortality in patients with sepsis (32, 33). In addition, an increased BUN/Cr ratio may reflect reduced effective circulating volume and stress-related hypercatabolism, and has been associated with poor outcomes in septic shock patients (17). These findings support the clinical relevance of renal and metabolic indicators in mortality prediction models for pediatric sepsis.

Lactate dehydrogenase (LDH) is a nonspecific marker of tissue injury and cellular metabolic disturbance. During sepsis, tissue hypoxia, inflammatory damage, and multiple organ dysfunction may lead to elevated LDH levels (34). Previous studies have suggested that increased LDH concentrations are associated with disease severity and unfavorable outcomes in critically ill patients (34). Therefore, LDH may provide additional information regarding the extent of systemic injury in pediatric sepsis.

INR is an important marker of coagulation dysfunction. During sepsis, inflammatory activation promotes coagulation cascade activation, resulting in coagulation factor consumption, impaired coagulation balance, and microvascular thrombosis, which may progress to disseminated intravascular coagulation in severe cases. The bidirectional interaction between inflammation and coagulation is considered a key mechanism underlying organ dysfunction and adverse outcomes in sepsis (35). Previous studies have shown that sepsis-associated coagulopathy assessed by coagulation parameters including INR is associated with disease severity and mortality risk (36). In our study, INR showed high importance in the machine learning model, suggesting that coagulation abnormalities may provide valuable information for mortality risk prediction in children with sepsis admitted to the PICU.

Our findings are consistent with recent studies showing the value of ensemble and boosting algorithms in clinical prediction tasks. Compared with meta-ensemble frameworks for early sepsis detection and advanced neural-network models developed for other diagnostic applications, the present study focused on a more clinically specific task: early mortality prediction in pediatric sepsis. The final model used only seven routinely available variables, which may improve its feasibility for bedside risk stratification. In addition, SHAP-based global and local explanations were incorporated to improve transparency, although these explanations should be interpreted as model-based predictive contributions rather than causal relationships.

Unlike traditional ‘black-box' machine learning models, this study used SHAP to interpret the final XGBoost model. Global SHAP analysis showed that INR, pH, LDH, PaO2, and Glu were among the variables with greater contributions to model predictions. These features represent coagulation function, acid-base status, tissue injury, oxygenation status, and metabolic status information captured by the model. However, SHAP importance represents the contribution of each variable to the model output and should not be interpreted as evidence of causal associations with mortality. Local SHAP waterfall plots further illustrated how individual variables contributed to a specific prediction, providing a transparent view of the model rationale for individual risk estimation.

Furthermore, this study developed a clinical application tool using Streamlit to enable real-time model prediction and risk stratification. Existing research indicates that deploying machine learning models as clinically accessible tools enhances their practical utility (37).

The probability threshold used in the primary analysis was selected using the Youden index, which provides a statistical balance between sensitivity and specificity. This threshold may be useful for exploratory risk stratification, but it should not be regarded as a universally optimal clinical cutoff. In pediatric sepsis, the clinical consequences of false-negative classification may be more serious when the model is used for early screening; therefore, a lower threshold may be preferred to improve sensitivity. Conversely, when the aim is to reduce false-positive alerts and unnecessary interventions, a higher threshold may be selected to improve specificity. Thus, the optimal threshold should be determined according to the intended clinical use and should be further validated in prospective clinical settings.

Although PRISM III and PELOD-2 achieved slightly higher AUC values, these established severity scores require substantially more clinical variables and were developed for comprehensive severity assessment rather than simplified individualized mortality prediction. Our model achieved competitive discrimination while requiring only seven routinely available variables and providing individualized explainability through SHAP analysis.

This study still has certain limitations. First, this was a single-center retrospective study with a relatively limited sample size, and external validation was not available (38). Although the model was developed using seven routinely available clinical variables collected within 24 h after PICU admission, which may facilitate implementation in other PICU settings, its generalizability remains uncertain because differences in patient characteristics, sepsis management strategies, laboratory measurement procedures, and data collection practices across institutions may affect model performance. Therefore, the current findings should be interpreted as internally validated results from a single center rather than evidence of confirmed external applicability. Although five-fold and ten-fold cross-validation and repeated stratified random split analyses were performed to assess model robustness, these internal validation approaches cannot replace independent external validation or prospective evaluation. Future studies will include temporal validation using more recent patient cohorts from our institution and multicenter external validation to further assess model transportability, calibration, and clinical utility. Second, the model was established based on variables within 24 h after PICU admission and did not include dynamic trends; future studies may further explore whether time-series features improve predictive performance (39). Third, although SHAP improved model interpretability, SHAP values explain the contribution of features to model predictions rather than causal relationships. The interpretation may also be influenced by correlations among predictors and unmeasured confounding. Accordingly, the model should be used to support, rather than replace, clinical judgment and should not serve as the sole basis for diagnosis or treatment decisions (39).

In summary, this study established a seven-variable XGBoost mortality risk prediction model for pediatric sepsis based on routine clinical indicators. The model showed good discrimination, a certain degree of stability, and interpretability, and can be converted into a convenient Web application tool.

In the future, temporal validation using later patient cohorts, multicenter external validation, and prospective clinical impact studies are needed to further evaluate the model's transportability, calibration, clinical utility, and effectiveness in supporting early risk stratification and clinical decision-making for pediatric sepsis.

## Data Availability

The datasets generated and/or analyzed during the current study are not publicly available because they contain identifiable patient information and are subject to institutional ethics and data protection regulations. Requests to access the datasets should be directed to the corresponding author and will be considered on reasonable request, subject to approval by the institutional ethics committee and applicable regulations. Requests to access the datasets should be directed to hua1970_sz@163.com.
