# Supplementary material for: Development and validation of an explainable XGBoost model for early mortality prediction in pediatric sepsis
Source: Front Pediatr. 2026 Aug 31;14:1933395. doi: 10.3389/fped.2026.1933395 (PMC13572637; doi:10.3389/fped.2026.1933395)
Supplement: Supplementary file 1 [file Datasheet1.pdf]

Table of contents

Supplementary Table S1. Training-set-only feature selection process. 2

Supplementary Table S2. Training-set-based VIF analysis for multicollinearity assessment. 3

Supplementary Table S3. Repeated 80:20 stratified random split sensitivity analysis of the final XGBoost model. 4

Supplementary Table S4. Class-imbalance sensitivity analyses of the final XGBoost model. 5

Supplementary Table S5. Comparison of the final XGBoost model with available clinical severity scores. 6

Supplementary Table S6. Calibration analysis of the final XGBoost model. 7

Supplementary Table S7. Threshold sensitivity analysis of the final XGBoost model. 8

Supplementary Table S8. Optimal hyperparameters of the 12 candidate machine learning models. 9

Supplementary Table S9. Python version, package dependencies, and random seed. 10

Supplementary Table S10. Bootstrap 95% confidence intervals for model performance metrics. 11

Supplementary Figure S1. Calibration curve of the final XGBoost model. 12

Supplementary Figure S2. Threshold sensitivity analysis of the final XGBoost model. 13

Supplementary Table S1. Training-set-only feature selection process.

Variable	Training_set_ROC_AUC	Decision
INR	0.737	Retained
AB	0.686	Retained
BE	0.683	Retained
Cr	0.675	Retained
Lac	0.671	Retained
APTT	0.667	Retained
pH	0.666	Retained
LDH	0.666	Retained
BUN	0.659	Retained
PaO2	0.631	Retained
ALB	0.627	Retained
Ca	0.619	Retained
Glu	0.606	Retained
SpO2	0.603	Retained
BUN_Cr_ratio	0.587	Retained
MAP	0.571	Retained
Na	0.568	Retained
PCO2	0.565	Retained
log1p_Cr	0.675	Removed
log1p_Lac	0.671	Removed
log1p_LDH	0.666	Removed
log1p_BUN	0.659	Removed
log1p_Glu	0.606	Removed
Resp	0.551	Removed
PCT	0.543	Removed
CRP	0.534	Removed
log1p_CRP	0.534	Removed
Hb	0.52	Removed
NeuPct_LymPct_ratio	0.519	Removed
NeuPct	0.512	Removed
Pulse	0.509	Removed
Plt	0.506	Removed
WBC	0.504	Removed

Note: For paired original and log(1+x)-transformed variables, the original variable was retained even when the transformed variable showed similar AUC, because the original scale has greater clinical interpretability and did not provide meaningful predictive improvement. Transformed variables were removed accordingly.

Supplementary Table S2. Training-set-based VIF analysis for multicollinearity assessment.

Variable	VIF	Iteration	Decision	Reason
BE	30.161	1	Removed	VIF > 10
AB	8.215	2	Retained	VIF < 10 after removal of high-collinearity variable
PCO2	6.746	2	Retained	VIF < 10 after removal of high-collinearity variable
pH	6.649	2	Retained	VIF < 10 after removal of high-collinearity variable
Cr	3.369	2	Retained	VIF < 10 after removal of high-collinearity variable
BUN	3.203	2	Retained	VIF < 10 after removal of high-collinearity variable
INR	2.06	2	Retained	VIF < 10 after removal of high-collinearity variable
APTT	1.704	2	Retained	VIF < 10 after removal of high-collinearity variable
Lac	1.496	2	Retained	VIF < 10 after removal of high-collinearity variable
BUN_Cr_ratio	1.442	2	Retained	VIF < 10 after removal of high-collinearity variable
ALB	1.341	2	Retained	VIF < 10 after removal of high-collinearity variable
Ca	1.314	2	Retained	VIF < 10 after removal of high-collinearity variable
Glu	1.285	2	Retained	VIF < 10 after removal of high-collinearity variable
PaO2	1.275	2	Retained	VIF < 10 after removal of high-collinearity variable
MAP	1.215	2	Retained	VIF < 10 after removal of high-collinearity variable
LDH	1.211	2	Retained	VIF < 10 after removal of high-collinearity variable
Na	1.164	2	Retained	VIF < 10 after removal of high-collinearity variable
SpO2	1.135	2	Retained	VIF < 10 after removal of high-collinearity variable

Note: VIF analysis was performed in the training set only. BE was removed because its VIF exceeded 10. After removal of BE, all remaining variables had VIF values below 10.

Supplementary Table S3. Repeated 80:20 stratified random split sensitivity analysis of the final XGBoost model.

Metric	Mean	SD	Median	2.5th_percentile	97.5th_percentile	N_repetitions
AUC	0.78	0.053	0.778	0.668	0.875	100
PR_AUC	0.584	0.079	0.587	0.424	0.746	100
Brier_score	0.106	0.011	0.106	0.084	0.128	100
sensitivity	0.672	0.128	0.654	0.423	0.885	100
specificity	0.823	0.106	0.836	0.579	0.984	100
accuracy	0.797	0.073	0.805	0.629	0.901	100
F1	0.542	0.072	0.535	0.41	0.701	100

Note: The repeated split analysis was performed 100 times using an 80:20 stratified random split. The final seven predictors and XGBoost hyperparameters were fixed across repetitions; only model fitting and validation were repeated.

Supplementary Table S4. Class-imbalance sensitivity analyses of the final XGBoost model.

Analysis	Threshold	AUC	PR_AUC	Brier_score	Sensitivity	Specificity
loaded_final_model	0.121	0.803	0.57	0.107	0.846	0.696
refit_without_weighting	0.116	0.801	0.572	0.108	0.846	0.696
refit_balanced_sample_weight	0.37	0.802	0.554	0.157	0.885	0.664
refit_scale_pos_weight	0.379	0.802	0.542	0.16	0.846	0.696

Note:

Balanced sample weighting and XGBoost scale_pos_weight were evaluated as class-imbalance sensitivity analyses. SMOTE was not used in the primary analysis because synthetic oversampling may generate artificial patient profiles and increase overfitting risk in modest-sized clinical datasets.

loaded_final_model refers to the final seven-feature XGBoost model selected in the primary analysis and loaded directly for evaluation in the internal validation cohort. refit_without_weighting refers to the same seven-feature XGBoost model refitted in the training cohort without applying any imbalance-handling strategy. refit_balanced_sample_weight refers to the model refitted using balanced sample weights to account for class imbalance. refit_scale_pos_weight refers to the model refitted using the XGBoost scale_pos_weight parameter, defined according to the ratio of non-survivors to survivors in the training cohort. The threshold was determined using the Youden index for each analysis. AUC, area under the receiver operating characteristic curve; PR-AUC, area under the precision–recall curve.

Supplementary Table S5. Comparison of the final XGBoost model with available clinical severity scores.

Model_or_score	AUC	AUC_95_CI	PR_AUC	PR_AUC_95_CI	N_bootstrap
Final XGBoost model	0.803	0.683-0.893	0.57	0.367-0.744	1000
PRISM3	0.828	0.707-0.932	0.683	0.497-0.843	1000
PELOD2	0.827	0.709-0.926	0.666	0.474-0.816	1000

Note: AUC and PR-AUC were calculated in the internal validation cohort. Confidence intervals were estimated using 1,000 bootstrap resamples. AUC, area under the receiver operating characteristic curve; PR-AUC, area under the precision–recall curve.

Supplementary Table S6. Calibration analysis of the final XGBoost model (Overall Brier Score = 0.107).

n_bins	bin	mean_predicted_probability	observed_mortality_rate
5	1	0.045	0.032
5	2	0.066	0.067
5	3	0.098	0.033
5	4	0.156	0.267
5	5	0.492	0.467
10	1	0.038	0.063
10	2	0.053	0.000
10	3	0.062	0.067
10	4	0.071	0.067
10	5	0.088	0.067
10	6	0.108	0.000
10	7	0.130	0.400
10	8	0.182	0.133

Note: Calibration was assessed in the internal validation cohort. Patients were grouped into quantile-based bins according to predicted mortality probability. The Brier score was 0.107; lower values indicate better agreement between predicted probabilities and observed outcomes.

Supplementary Table S7. Threshold sensitivity analysis of the final XGBoost model.

threshold	AUC	sensitivity	specificity	PPV	NPV	accuracy	F1	Youden_index
0.05	0.803	0.962	0.136	0.188	0.944	0.278	0.314	0.098
0.1	0.803	0.846	0.576	0.293	0.947	0.623	0.436	0.422
0.121	0.803	0.846	0.696	0.367	0.956	0.722	0.512	0.542
0.15	0.803	0.615	0.800	0.390	0.909	0.768	0.478	0.415
0.2	0.803	0.577	0.840	0.429	0.905	0.795	0.492	0.417
0.3	0.803	0.423	0.920	0.524	0.885	0.834	0.468	0.343
0.4	0.803	0.385	0.952	0.625	0.881	0.854	0.476	0.337
0.5	0.803	0.346	0.960	0.643	0.876	0.854	0.450	0.306

Note: The Youden-index threshold was 0.121. Lower thresholds prioritize sensitivity and may be preferred for screening scenarios, whereas higher thresholds prioritize specificity and may reduce false-positive alerts. PPV, positive predictive value; NPV, negative predictive value. For clinical application, a lower threshold (e.g., 0.05–0.10) may be preferred in screening scenarios to maximize sensitivity and avoid missing high-risk patients, whereas a higher threshold (e.g., ≥ 0.30) may be used when confirmatory testing is limited to reduce false-positive referrals.

Supplementary Table S8. Optimal hyperparameters of the 12 candidate machine learning models.

Model	Estimator_class	Best_CV_AUC	Optimal_hyperparameters
ADABOOST	AdaBoostClassifier	0.731	{"learning_rate": 0.03, "n_estimators": 100}
ANN	Pipeline	0.7	{"model__alpha": 0.01, "model__hidden_layer_sizes": [32]}
DT	DecisionTreeClassifier	0.729	{"ccp_alpha": 0.0, "max_depth": 2, "min_samples_leaf": 12, "min_samples_split": 2}
EXT	ExtraTreesClassifier	0.794	{"max_depth": null, "max_features": 0.4, "min_samples_leaf": 4, "min_samples_split": 10}
GBM	GradientBoostingClassifier	0.77	{"learning_rate": 0.1, "max_depth": 3, "min_samples_leaf": 5, "n_estimators": 100, "subsample": 0.7}
GLM	Pipeline	0.759	{"model__C": 10}
KNN	Pipeline	0.759	{"model__n_neighbors": 11, "model__weights": "distance"}
LIGHTGBM	LGBMClassifier	0.779	{"colsample_bytree": 0.8, "learning_rate": 0.01, "max_depth": 5, "min_child_samples": 10, "n_estimators": 50, "num_leaves": 15, "reg_lambda": 5, "subsample": 0.8}
NB	GaussianNB	0.763	{"var_smoothing": 1e-09}
RF	RandomForestClassifier	0.795	{"max_depth": null, "max_features": "sqrt", "min_samples_leaf": 4, "min_samples_split": 2}
SVM	Pipeline	0.784	{"model__C": 10, "model__gamma": 0.01}
XGBOOST	XGBClassifier	0.79	{"colsample_bytree": 1.0, "learning_rate": 0.03, "max_depth": 2, "min_child_weight": 5, "n_estimators": 200, "reg_lambda": 5, "subsample": 0.7}

Note: Hyperparameters were selected using 5-fold stratified cross-validation with grid search in the training set, with ROC-AUC as the optimization metric. The final selected model was XGBoost.

Supplementary Table S9. Python version, package dependencies, and random seed.

Software_or_package	Version
Python	3.10.11
platform	Windows-10-10.0.19045-SP0
numpy	2.2.6
pandas	2.3.3
scikit-learn	1.7.2
xgboost	3.2.0
lightgbm	4.6.0
shap	0.49.1
matplotlib	3.10.9
seaborn	0.13.2
scipy	1.15.3
statsmodels	0.14.6
joblib	1.5.3
streamlit	1.57.0
global_random_seed	20251112

Note: Package versions and the global random seed are reported to facilitate computational reproducibility. The global random seed was 20251112.

Supplementary Table S10. Bootstrap 95% confidence intervals for model performance metrics.

Model or predictor	Metric	Estimate	95% CI
Final seven-feature XGBoost	AUC	0.803	0.683-0.893
Final seven-feature XGBoost	PR_AUC	0.57	0.367-0.744
Final seven-feature XGBoost	Brier_score	0.107	0.074-0.146
Final seven-feature XGBoost	sensitivity	0.846	0.692-0.964
Final seven-feature XGBoost	specificity	0.696	0.619-0.771
Final seven-feature XGBoost	accuracy	0.722	0.649-0.788
Final seven-feature XGBoost	F1	0.512	0.369-0.633
INR	AUC	0.717	0.578-0.833
INR	PR-AUC	0.464	0.255-0.651
BUN	AUC	0.671	0.561-0.778
BUN	PR-AUC	0.268	0.167-0.435
LDH	AUC	0.667	0.546-0.778
LDH	PR-AUC	0.378	0.203-0.548
pH	AUC	0.661	0.532-0.787
pH	PR-AUC	0.389	0.210-0.574
PaO2	AUC	0.656	0.537-0.765
PaO2	PR-AUC	0.254	0.147-0.405
Glu	AUC	0.593	0.441-0.730
Glu	PR-AUC	0.306	0.163-0.502
BUN_Cr_ratio	AUC	0.509	0.380-0.625
BUN_Cr_ratio	PR-AUC	0.177	0.106-0.294
PRISM3	AUC	0.828	0.707-0.932
PRISM3	PR-AUC	0.683	0.497-0.843
PELOD2	AUC	0.827	0.709-0.926
PELOD2	PR-AUC	0.666	0.474-0.816

Note: Confidence intervals were estimated using 1,000 bootstrap resamples of the internal validation cohort. Classification metrics were calculated using the Youden-index threshold identified for the final model. AUC, area under the receiver operating characteristic curve; PR-AUC, area under the precision–recall curve; CI, confidence interval.

Supplementary Figure S1. Calibration curve of the final XGBoost model.

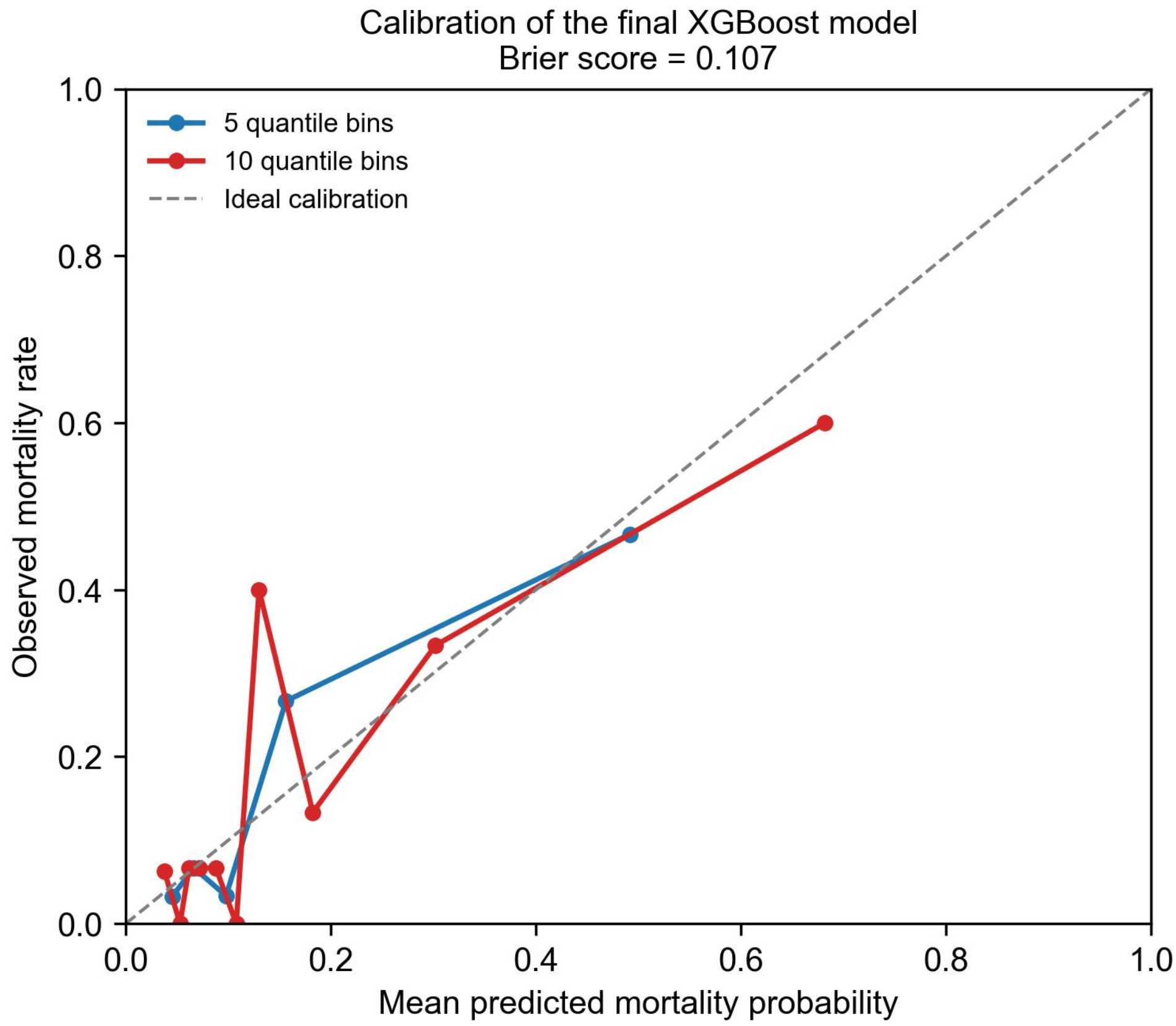

Note: Calibration was assessed in the internal validation cohort. Patients were grouped into quantile-based bins according to predicted mortality probability. The Brier score was 0.107; lower values indicate better agreement between predicted probabilities and observed outcomes.

Supplementary Figure S2. Threshold sensitivity analysis of the final XGBoost model.

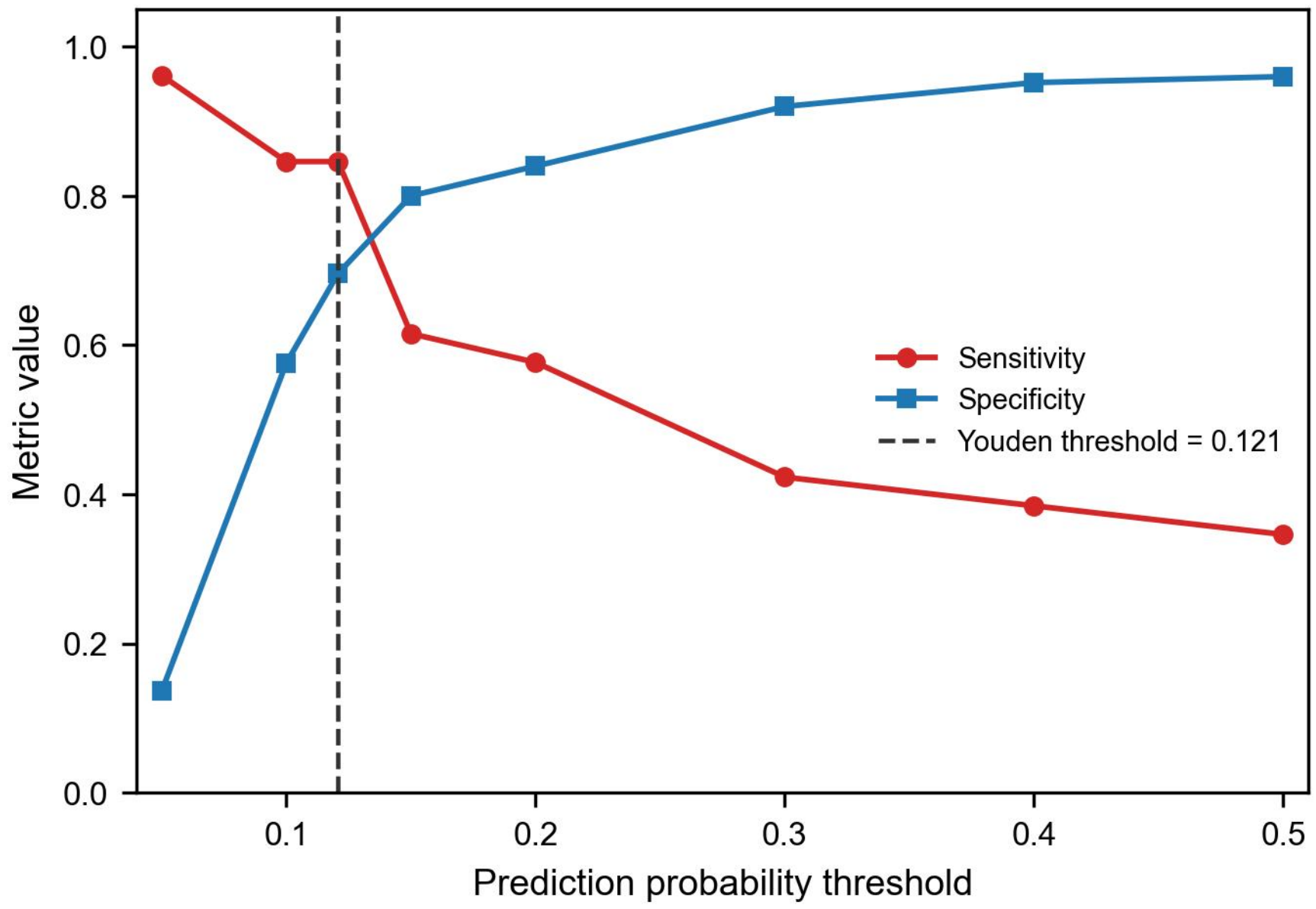

Note: Sensitivity decreased and specificity increased as the prediction probability threshold increased. The dashed vertical line indicates the optimal threshold determined by the Youden index.
